# Altered Vascular Endothelium-Dependent Responsiveness in Frail Elderly Patients Recovering from COVID-19 Pneumonia: Preliminary Evidence

**DOI:** 10.3390/jcm10122558

**Published:** 2021-06-09

**Authors:** Mara Paneroni, Evasio Pasini, Michele Vitacca, Simonetta Scalvini, Laura Comini, Anna Pedrinolla, Massimo Venturelli

**Affiliations:** 1 Respiratory Rehabilitation of the Institute of Lumezzane, Istituti Clinici Scientifici Maugeri IRCCS, 25065 Lumezzane, Italy; michele.vitacca@icsmaugeri.it; 2 Cardiac Rehabilitation of the Institute of Lumezzane, Istituti Clinici Scientifici Maugeri IRCCS, 25065 Lumezzane, Italy; evasio.pasini@icsmaugeri.it (E.P.); simonetta.scalvini@icsmaugeri.it (S.S.); 3 Scientific Direction of the Institute of Lumezzane, Istituti Clinici Scientifici Maugeri IRCCS, 25065 Lumezzane, Italy; laura.comini@icsmaugeri.it; 4 Section of Movement Science, Department of Neuroscience, Biomedicine, and Movement Science, University of Verona, 37100 Verona, Italy; anna.pedrinolla@univr.it (A.P.); massimo.venturelli@univr.it (M.V.); 5 Section of Geriatrics, Department of Internal Medicine, University of Utah, Salt Lake City, UT 84132, USA

**Keywords:** inflammation, endothelial dysfunction, nitric oxide, COVID-19, single leg passive movement

## Abstract

We evaluated vascular dysfunction with the single passive leg movement test (sPLM) in 22 frail elderly patients at 84 + 31 days after hospitalization for COVID-19 pneumonia, compared to 22 age-, sex- and comorbidity-matched controls (CTRL). At rest, all COVID-19 patients were in stable clinical condition without severe comorbidities. Patients (aged 72 ± 6 years, 73% male) had moderate disability (Barthel index score 77 ± 26), hypoxemia and normocapnia at arterial blood gas analysis and mild pulmonary restriction at spirometry. Values of circulating markers of inflammation (C-reactive protein: CRP; erythrocyte sedimentation rate: ESR) and coagulation (D-dimer) were: 27.13 ± 37.52 mg/dL, 64.24 ± 32.37 mm/1 h and 1043 ± 729 ng/mL, respectively. At rest, femoral artery diameter was similar in COVID-19 and CTRL (*p* = 0.16). On the contrary, COVID-19 infection deeply impacted blood velocity (*p* = 0.001) and femoral blood flow (*p* < 0.0001). After sPLM, peak femoral blood flow was dramatically reduced in COVID-19 compared to CTRL (*p* = 0.001), as was blood flow ∆peak (*p* = 0.05) and the area under the curve (*p* < 0.0001). This altered vascular responsiveness could be one of the unknown components of long COVID-19 syndrome leading to fatigue, changes in muscle metabolism and fibers’ composition, exercise intolerance and increased cardiovascular risk. Impact of specific treatments, such as exercise training, dietary supplements or drugs, should be evaluated.

## 1. Introduction

Coronavirus disease 2019 (COVID-19) is a worldwide pandemic that has infected patients in more than 200 countries around the world [1].

COVID-19 is caused by the SARS-CoV-2 virus, which infects the cells of many organs through the human angiotensin-converting enzyme 2 (ACE2) receptor with consequent organ/system dysfunction [2]. ACE2 is expressed in the lung, heart, kidneys, intestinal epithelium and vascular endothelium [3], which has been demonstrated to be directly infected by COVID-19 [4]. Notably, the endothelium produces molecules such as nitric oxide (NO) that are fundamental to regulating cardiovascular system functions. Consequently, impaired endothelial function has an important clinical impact in relation to altered vascular tone, inflammation and coagulation. These latter phenomena are particularly evident in elderly patients who have the highest COVID-19-related mortality rate, predominantly linked to an accentuated vasoconstriction and pro-coagulative state, probably endothelium-mediated [5,6,7].

In light of these observations, the European Society of Cardiology (ESC) has recently pointed out the importance of evaluating vascular endothelial function in patients with COVID-19 [8].

Among the available vascular function tests, passive leg movement (PLM) and its shorter version, single passive leg movement (sPLM), which assess the change in femoral artery blood flow in response to the passive movement of the leg, provide a simple, repeatable, reliable and specific index of vascular endothelial function predominantly as a consequence of NO-mediated vasodilation [9]. Indeed, sPLM was recently used on COVID-19 patients by Ratchford at al [10], who found significant endothelial dysfunction in young adults positive for SARS-CoV-2.

However, the proportion of young adults impacted by COVID-19 is marginal and the risk of vascular sequelae is higher in frail elderly. Therefore, this study aimed to evaluate whether frail elderly recovering from COVID-19 pneumonia have altered vascular endothelium-dependent responsiveness.

## 2. Materials and Methods

This is a cross-sectional comparative study performed on 22 patients at 84 ± 31 days after acute care hospitalization for documented COVID-19 pneumonia (COVID-19). Patients were recovering from COVID in the rehabilitation unit of the Clinical and Scientific Institutes (ICS) Maugeri of Lumezzane (Brescia), Italy, where the study was carried out between July and October 2020. Data were compared to those of a Control group (CTRL) consisting of 22 sex-, age- and comorbidity-matched volunteers recruited at the Department of Neuroscience, Biomedicine and Movement of the University of Verona.

Inclusion criteria were: post-COVID patients aged > 50 years, in stable clinical condition (temperature < 37.5 °C, respiratory rate (RR) < 22 breaths/min, heart rate (HR) > 50 beats/minute and <120 beats/minute, absence of major arrhythmias, hemodynamic stability), and able to sit down independently. Written, informed consent was obtained from all participants before inclusion in the study. The Ethics Committee approved the study (2437-Ethical Committee; 19 June 2020), and all experimental procedures were performed in accordance with the Declaration of Helsinki.

### 2.1. Study Overview

#### 2.1.1. Data Collection

At enrollment (i.e., after acute care hospitalization) baseline anthropometrical and clinical data were collected. Comorbidities were assessed with the cumulative illness rating scale (CIRS) [11].

Respiratory evaluations included arterial blood gas (ABG) analysis (i.e., PaO_2_, PaCO_2_ and pH performed at room temperature) and spirometry (forced volume capacity (FVC)% of predicted, forced expiratory volume at 1 s (FEV_1_) % of predicted and FEV_1_/FVC) [12,13].

The values of circulating markers of inflammation (C-reactive protein: CRP; erythrocyte sedimentation rate: ESR) and coagulation (D-dimer) were analyzed in the blood at rest.

#### 2.1.2. Systemic Vascular Function via sPLM

The sPLM test was performed on the right common femoral artery, and measurements were made using a Doppler ultrasound system (Logiq V4-GE, Milwaukee, WI, USA). Arterial blood velocity and vessel diameter were measured in the passively moved leg, distal to the inguinal ligament and proximal to the deep and superficial femoral bifurcation. The sPLM protocol consisted of 60 s of resting baseline data collection, followed by a passive knee flexion and extension of 1 s. The leg was then maintained fully extended for the remaining 60 s after movement.

Resting arterial diameter, resting blood flow, relative changes (∆peak) from rest, peak blood flow and the area under the curve (AUC) of femoral blood flow were determined for each subject. Arterial diameter was measured as the distance (mm) between the intima–lumen interfaces for the anterior and posterior walls in the common femoral artery. Leg blood flow was calculated using arterial diameter blood velocity according to the formula:

Blood Flow = [Vmean (mean of Blood Velocity) × π (vessel diameter/2)^2^ × 60 (mL/min)]

The values of peak blood flow, relative changes from rest (∆peak) and AUC after the leg movement were calculated second-by-second. During the test, subjects rested in an upright-seated position for 20 min before the start of data collection and remained in this position throughout this part of the study [14].

#### 2.1.3. Level of Physical Function

In all patients, disability and physical performance status were assessed with the Barthel index [15], short physical performance battery (SPPB) [16,17] and the 6-minute walking test (6-MWT) [18].

### 2.2. Data Analysis

Data were expressed as mean ± standard deviation. A Student’s unpaired T test was used to establish differences between groups at rest and after the passive movement. Two–way ANOVA was used to analyze the hyperemic response to sPLM in COVID-19 versus CTRL (interaction group × time). Significance was set at an α level of 0.05.

## 3. Results

Clinical characteristics of the COVID-19 patients and control subjects at enrollment are illustrated in Table 1. Patients were mainly male (73%) and had normal body mass index (BMI), moderate disability (Barthel index) and severe exercise intolerance (6-MWT). Patients with COVID-19 were hypoxemic and normocapnic at ABG analysis, with mild pulmonary restriction at spirometry (FEV1 71 ± 23% and FCV 63 ± 23). During the acute hospital phase, all patients had been on oxygen therapy, and about half of them underwent invasive ventilation. About one-quarter of the patients had a clinical history of pulmonary embolism and pneumothorax. The values of circulating markers of inflammation (CRP and ESR) and coagulation (D-dimer) were all altered with respect to normal values, suggesting that the inflammatory and coagulative system was highly activated in these patients (Table 1).

**Figure 1:**
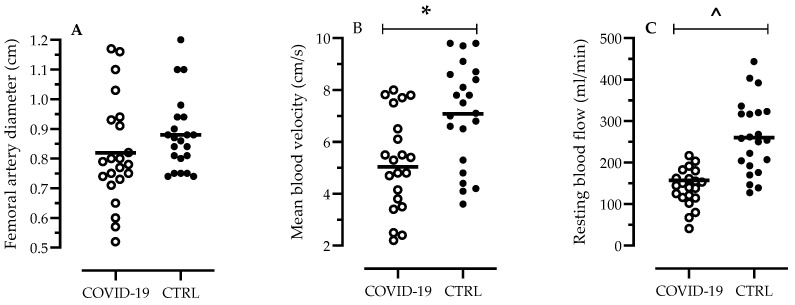
Femoral artery diameter (**A**), blood velocity (**B**) and femoral blood flow (**C**), at rest in COVID-19 patients (*n* = 22) versus age- and sex-matched controls (CTRL, *n* = 22). Data are presented in a scatter plot, and black bars indicate the mean value; * refers to *p* = 0.001, ^ refers to *p* < 0.001.

The evaluation of femoral artery diameter by echo-scan was similar in COVID-19 and CTRL (0.81 ± 0.18 cm versus 0.89 ± 0.12 cm; *p* = 0.16) (Figure 1A). On the contrary, COVID-19 infection deeply impacted blood velocity (5.0 ± 1.9 cm/s versus 7.1 ± 1.9 cm/s; *p* = 0.001) (Figure 1B) and femoral blood flow (157 ± 89 mL/min versus 265 ± 88 mL/min; *p* <0.0001) in the resting state (Figure 1C).

**Figure 2:**
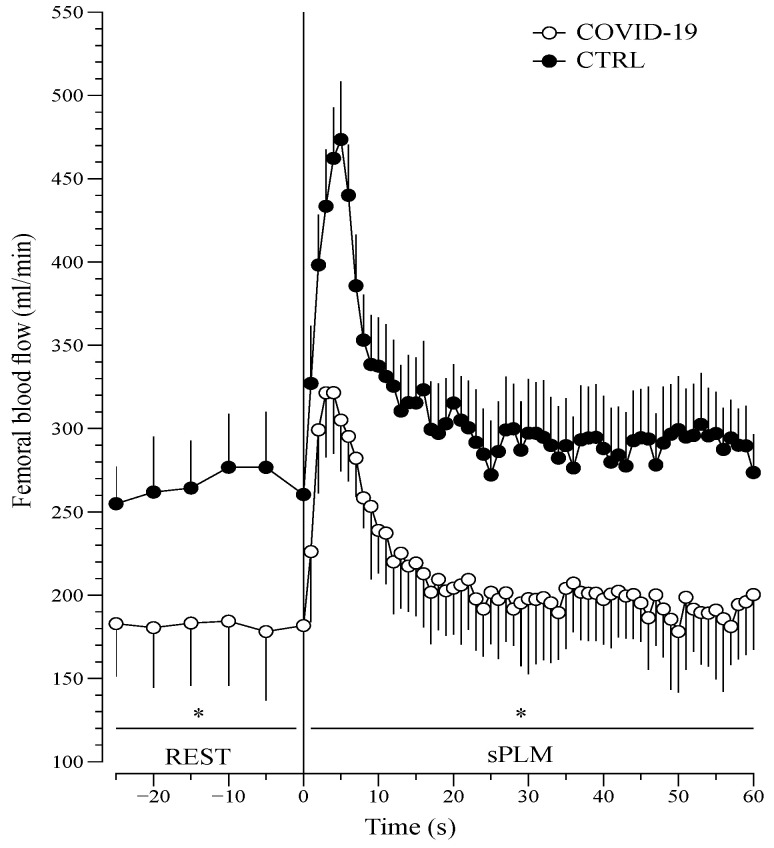
Hyperemic response to sPLM in COVID-19 and CTRL. Legend: Femoral blood flow in response to single passive leg movement in patients with COVID-19 (*n* = 22) as well as in age- and sex-matched controls (CTRL, *n* = 22). Data are presented as mean ± standard error (SE). * *p* = 0.001, interaction of group × time (2-way ANOVA).

The femoral blood flow change during the sPLM evaluation (Figure 2) shows that the sPLM-induced hyperemia response was drastically reduced in patients with COVID-19 (*p* = 0.001).

**Figure 3:**
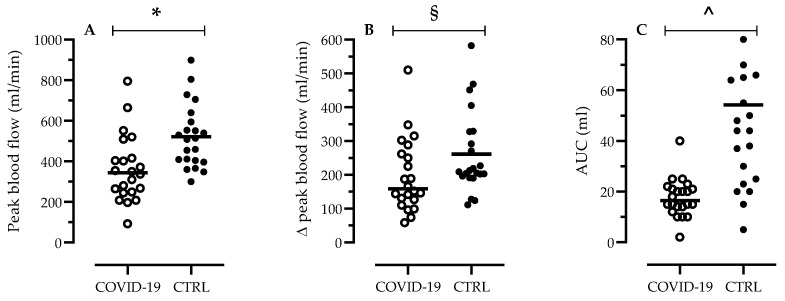
Peak blood flow (**A**), the relative changes from rest (∆peak, **B**) and the area under the curve (AUC, **C**) during the sPLM in COVID-19 patients (*n* = 22) versus age- and sex-matched controls (CTRL, *n* = 22). Data are presented in a scatter plot, and black bars indicate the mean value; § refers to *p* = 0.05; * refers to *p* = 0.001, ^ refers to *p* < 0.001.

Figure 3 shows the values of peak blood flow (Figure 3A), the relative changes from rest (∆peak, panel B) and the AUC (panel C) during the test in the two groups. The peak of the femoral blood flow, reached after ~5 s of sPLM, differed significantly between COVID-19 and CTRL (364 ± 165 mL/min versus 529 ± 156 mL/min, *p* = 0.001) (Figure 3A). Interestingly, also the relative blood flow change from baseline differed significantly between COVID-19 and CTRL (196 ± 108 mL/min versus 265 ± 123 mL/min, *p* = 0.05) (Figure 3B). In line with the peak values of blood flow, also the AUC (total volume of blood following the sPLM) was significantly decreased in patients with COVID-19 compared to CTRL (*p* < 0.0001; Figure 3C).

## 4. Discussion

This is the first study to show an altered vascular endothelium-dependent responsiveness in frail elderly recovering from pneumonia at 2–4 months from COVID-19 infection. The endothelial dysfunction is concomitant with the presence of systemic inflammation and a hypercoagulable state. The altered vascular responsiveness found in our study could be due to the direct and/or indirect actions of SARS-CoV-2.

It is well known that SARS-CoV-2 uses human ACE2 as an entry receptor to infect host cells, and ACE2 receptors are present in endothelial cells. Hence, the virus can directly infect the endothelium [4]. The ACE2 receptor intracellular pathway is very complex and regulates local renin-angiotensin system (RAS) peptides, fundamental for maintaining endothelium metabolism. Thus, local RAS dysregulation due to SARS-CoV-2 infection can alter vasculature functions [19].

Endothelium synthesizes and releases many molecules, e.g., prostaglandin, endothelin, thromboxane A2, von Willebrand factor (vWF) and NO, which regulate both vascular functions and platelet aggregation and adhesion [20]. Moreover, there is clear evidence that endothelial-derived molecules, such as NO, modulate leucocyte activity, reducing local inflammation and consequent cell damage [21].

Hence, the local SARS-CoV-2-induced endothelial inflammation and consequent dysfunction may be due to different mechanisms. Indirectly, the endothelial dysfunction observed in our patients via sPLM could be due, at least in part, to systemic inflammation caused by the SARS-CoV-2-induced cytokine storm. In fact, endothelium belongs to the innate immune system and is one of the frontline physical barriers. Interestingly, endothelial cells play an important role in activating the innate immune response through specific receptors, such as Toll-like receptors (TLRs) [22]. Notably, single-stranded RNA from SARS-CoV-2 binds to TLRs 3, 7, 8 and 9 and turns on signal–transduction messages able to stimulate nuclear factor kappa-B (NF-kB) and mitogen-activated protein kinase (MAPK). These latter activate a specific intracellular cascade promoting the production of pro-inflammatory interleukins (IL-6, IL-8, TNF and IL-1B) and adhesion molecules (ICAM, E-selectin) triggering the synthesis of pro-coagulants and regulatory vascular tone molecules, such as vWF or plasminogen activator inhibitor type-1 (PAI-1), which cause altered vascular responsiveness [23].

It is well established that inflammation generates reactive oxygen species (ROS), which react with NO reinforcing endothelial dysfunction predominantly by reducing NO bioavailability. ROS could be produced also by the SARS-induced alteration of endothelial mitochondrial enzymes, such as NADPH oxidase [24].

Notably, our results are in line with the presence of endothelial dysfunction found in young patients two weeks after SARS-CoV-2 infection [10].

However, it is well known that obesity, diabetes and hypertension are the most prevalent diseases in elderly people and that endothelial dysfunction plays a fundamental role in the pathogenesis of these diseases. Interestingly, clinical data show that the incidence of angiopathy with pulmonary embolism and deep vein thrombosis is higher in elderly SARS-CoV-2-induced pneumonia patients with age-related comorbidities and that these comorbidities influence the patient’s prognosis, suggesting that SARS-CoV-2-induced endothelial damage reinforces the pre-existing endothelial dysfunction associated with age-related diseases [25,26].

More importantly, our data show that global inflammation (CRP) and a hypercoagulable state (D-dimer) were present in concomitance with endothelial dysfunction and that these alterations still persisted at 2–4 months from the onset of the acute infection.

Additionally, other factors may have influenced the development of endothelial dysfunction in this population. First, the bed-rest experienced by the patients during their long-term hospitalization. For instance, a previous study [27] described the reduction of peripheral blood flow and endothelial function due to inactivity, which is in line with our finding. Second, the advanced age of the patients may have influenced our results, which may reflect the natural age-related reduction of endothelial function [28].

We believe that our findings have important clinical implications. Identification of an altered endothelial-mediated vascular function by a simple and non-invasive test such as sPLM in combination with blood markers of inflammation and activated coagulation is important in order to stratify COVID-19 patients according to their cardiovascular risk, to allow tailored treatments to preserve vascular function and to counteract blood embolism and inflammatory hypercatabolism. The ESC recently drew attention to these clinical aspects, and stated that measuring endothelial function, in addition to myocardial injury and respiratory function markers in convalescent patients, may be a possible means for the early detection of vascular post COVID-19 sequelae.

This endothelial dysfunction could potentially be one of the not yet known components of long COVID-19 syndrome [29]: the reduction in vasodilation during exertion could produce fatigue, changes in muscle metabolism and fiber composition, and exercise intolerance.

This study could also open the discussion about possible treatments of this dysfunction: it is known that physical exercise can improve endothelial function [30,31], and rehabilitation attempts could lead to a beneficial improvement as well. Other treatments that could produce improvement are specific classes of dietary supplements [32] or drugs [33,34,35]. 

### Limitations

The main limitations of our study are: (a) the presence of comorbidities in COVID-19 patients, which could have had a previous impact on endothelial function. However, we compared COVID-19 patients with a group of matched subjects with the same presence of comorbidities; thus, we can hypothesize that the reduction in blood flow is mainly due to the recent COVID infection and its clinical consequences; (b) our study may suffer from low external validity as our post COVID-19 patients had a long hospital stay and high disability needs; (c) we did not evaluate vascular conductance, and we cannot provide any information on sympathetic vasoconstrictor nerve activity; moreover, (d) patients took several drug therapies that would have influenced our results, although we found no differences between patients who took drugs compared to who did not; (e) the vasodilation induced by the sPLM maneuver found in our study could be partially NO-related, and we cannot exclude that the observed alteration in vascular function might be, in part, SMCs-dependent [36,37].

## 5. Conclusions

Our study shows that NO-mediated vascular dysfunction together with inflammation and a hypercoagulable state are still present in frail elderly 2–4 months after COVID-19 pneumonia infection. Further specific follow-up studies are warranted to evaluate these alterations, using the simple and repeatable test here proposed, in order to identify long-term vascular alterations following recovery from COVID-19. If our data are confirmed by further studies, it will be important to implement a tailored therapy for endothelial protection in elderly ‘long COVID-19′ patients.

## Figures and Tables

**Table 1:** Clinical characteristics of COVID-19 patients and controls (CTRL).

	COVID-19 Patients (*n* = 22)	CTRL (*n* = 22)	*p*
Age, years	72.6 ± 8.7	72.5 ± 8.5	0.9694
Sex, male%	72.7	68.2	0.7436
BMI, Kg/m^2^	24.7 ± 3.3	24.6 ± 1.8	0.9013
Barthel index, score	76.6 ± 25.7	98.1 ± 3.7	0.0004
6-MWT, meters	195.4 ± 117.1	545 ± 135.5	<0.001
SPPB, score	5.8 ± 3.7	10.7 ± 0.6	<0.001
Comorbidities, %			
Hypertension	57	56	0.9467
Diabetes	19	30	0.3963
Heart failure	26	19	0.5782
Ischemic Heart Disease	17	10	0.4969
Pulmonary Chronic Disease	39	28	0.4395
CIRS1, score	2.2 ± 0.6		
CIRS2, score	5.8 ± 3.1		
PaO_2_, mmHg	61.2 ± 8.5	Normal value: >85 mmHg	
PaCO_2_, mmHg	42.3 ± 3.6	Normal value: 37–43 mmHg	
pH	7.43 ±0.01	Normal value: 7.37–7.44	
FEV_1_, %	71 ± 23	Normal value: >80%	
FVC, %	63 ± 23	Normal value: 80–120%	
FEV_1_/FVC	89 ± 10	Normal value: 80	
CRP, mg/dl	27.13 ± 37.52	Normal Value: ≤5 mg/L	
ESR, mm/h	64.24 ± 32.37	Normal value: W ≤ 30 mm/h M ≤ 20 mm/h	
	47.33 ± 25.06		
	73.45 ± 30.65		
D-dimer, ng/ml	1043 ± 729	Normal value: ≤300 ng/mL	
Clinical History, %			
Mechanical ventilation use	41		
Oxygen	100		
Embolism	23		
Pneumothorax	23		
Drugs, %			
Anti-rheumatic	5		
Cortisone	30		
Anti-malarial	35		
Anti-viral	25		
Anti-platelet	53		

Legend: Body mass index: BMI; 6-minute walking test: 6-MWT; short physical performance battery: SPPB; C-reactive protein: CRP; erythrocyte sedimentation rate: SR; cumulative illness rating scale: CIRS; arterial oxygen partial pressure: PaO_2_; arterial carbon dioxide partial pressure: PaCO_2_; forced expiratory volume at first second: FEV_1_; forced vital capacity: FV; W: women; M: men.

## Data Availability

Data are available close to corresponding author.
